# Comprehensive profiling of lysine ubiquitome reveals diverse functions of lysine ubiquitination in common wheat

**DOI:** 10.1038/s41598-017-13992-y

**Published:** 2017-10-19

**Authors:** Ning Zhang, Lingran Zhang, Chaonan Shi, Qiuzhen Tian, Guoguo Lv, Ying Wang, Dangqun Cui, Feng Chen

**Affiliations:** Agronomy College/National Key Laboratory of Wheat and Maize Crop Science/Collaborative Innovation Center of Henan Grain Crops, Henan Agricultural University, Zhengzhou, 450002 China

## Abstract

Protein ubiquitination, which is a major post-translational modifications that occurs in eukaryotic cells, is involved in diverse biological processes. To date, large-scale profiling of the ubiquitome in common wheat has not been reported, despite its status as the major cereal crop in the world. Here, we performed the first ubiquitome analysis of the common wheat (*Triticum aestivum* L.) variety, Aikang 58. Overall, 433 lysine modification sites were identified in 285 proteins in wheat seedlings, and four putative ubiquitination motifs were revealed. In particular, 83 of the 285 ubiquitinated proteins had ubiquitination orthologs in *Oryza sativa* L., and *Arabidopsis thaliana*. Ubiquitylated lysines were found to have a significantly different preference for secondary structures when compared with the all lysines. In accordance with previous studies, proteins related to binding and catalytic activity were predicted to be the preferential targets of lysine ubiquitination. Besides, protein interaction network analysis reveals that diverse interactions are modulated by protein ubiquitination. Bioinformatics analysis revealed that the ubiquitinated proteins were involved in diverse biological processes. Our data provides a global view of the ubiquitome in common wheat for the first time and lays a foundation for exploring the physiological role of lysine ubiquitination in wheat and other plants.

## Introduction

It is now abundantly clear that plant proteins are subjected to a wide array of post-translational modifications (PTMs) that greatly expand proteome functionality from more limited genomic information^1^. Among over 300 possibilities^2^, the majority of studies of PTMs on a proteomic scale in crops have focused on a small number of the most common types, such as phosphorylation, methylation, acetylation, glycosylation, ubiquitination, carbonylation and nitrosylation. Ubiquitin (Ub) is a protein composed of 76 amino acids found in both the cytosol and the nucleus of eukaryotic cells^3,4^. Protein degradation facilitated by the ubiquitin-proteasome system (UPS) is a major contributor to proteome remodeling^5^. In this pathway, ubiquitination occurs via the combination of Ub in lysine (Lys) residues of the acceptor proteins^6^. The process of ubiquitination typically leads to formation of an amide linkage comprising the ε-amine of lysine (Lys) of the target protein and the C terminus of Ub and can involve ubiquitination at distinct sites within the same protein^7^. This process occurs through a well-known enzymatic cascade involving E1 ubiquitin-activating enzymes, E2 ubiquitin-conjugating enzymes, and E3 ubiquitin ligases^4,6^. Many studies have shown that ubiquitination also mediates many other cellular processes involved in growth and development of plants, such as embryogenesis, photomorphogenesis and hormone regulation, as well take part in immune/stress responses, membrane transport, DNA repair, chromatin remodeling, and transcriptional regulation^2,4,8–10^.

Proteome-wide approaches are desirable to obtain a global understanding of the role of ubiquitination. Hundreds or thousands of targets have been reported based on proteomic approaches via mass spectrometry (MS) analyses of yeast^11^ and mammalian cells^12–14^; however, only a relatively small number of ubiquitination sites were mapped although many ubiquitinated proteins were identified^12^, This is because the methods in these studies required enrichment of ubiquitinated proteins^15^. Accordingly, more robust methods of ubiquitylation site identification and quantification are needed. After the ubiquitinated proteins undergoes proteolytic digestion with trypsin, the diglycine (di-Gly) remnant derived from the two C-terminal glycine residues of ubiquitin remains covalently linked to the modified lysines. Thus, antibodies recognizing the di-Gly remnant on lysine residues enable the affinity capture of ubiquitinated peptides^15^. The di-Gly modification causes a mass shift (114.0429 Da) of the parent peptide, which enables identification of the precise location of ubiquitination sites based on peptide fragment masses^15^. Several proteome-wide in-depth ubiquitination analyses in mammalian cells were recently conducted based on di-Gly-Lys-specific antibody enrichment^5,7,15^, which led to identification of a large number of ubiquitination sites. In addition, the availability of this method has also been tested in plants (rice)^16^.

Common wheat (*Triticum aestivum* L.), also known as bread wheat, is one of the most important cereal crops in the world. In China, the Yellow and Huang wheat region is a major wheat-producing area, accounting for 60–70% of the national output. Because of its excellent drought resistance, disease resistance and resistance to frost, Aikang 58 has been the most popular cultivar in the Yellow and Huang wheat region for several years. Even though the ubiquitomes in *Arabidopsis* ^1,17–20^ and rice (*Oryza sativa*)^16,21^ have been studied, the proteome-wide ubiquitination data are still lacking for other plant species. To date, the proteome-wide identification of lysine-ubiquitinated proteins has not been accomplished in wheat. Elucidation of the ubiquitome in wheat cells is important for understanding the role of the UPS in regulating development and stress responses. In this study, we performed an overall profiling of the ubiquitome of leaves of two-leaf stage wheat seedlings using integrated proteomic techniques in which ubiquitylated peptides are directly enriched from a trypsin-digested whole wheat cell peptide mixture with a commercially high affinity anti-di-Gly-Lys-specific antibody^7^. We then analyzed lysine ubiquitination sites in wheat for the first time via highly sensitive MS and bioinformatics tools. A total of 433 lysine modification sites were identified on 285 proteins in wheat seedlings, controlling various biological processes such as signal transduction, transport, metabolism, and response to stimulus. These ubiquitinated proteins are localized in multiple compartments, mainly cell, membrane, and organelle. This study provides a global view of this important cereal crop ubiquitome and an abundant dataset for examination of functions of Ub-related proteins in wheat.

## Materials and Methods

### Plant material and growth conditions

The seeds of wheat cultivar Aikang58 were immersed and sterilized with 1% (w/v) H_2_O_2_ for 0.5 h, then were thoroughly washed with distilled water. The sterilized seeds were covered with water in Petri dishes for 24 h to germinate, after which they were grown in one-half strength Murashige and Skoog medium. The uniform seedlings (≈4.1 ± 0.03 cm) were transferred into plastic pots with soil for growth. The wheat seedlings were kept in an illuminated incubator (RTOP-1000D, Zhejiang, China) at 25 °C/15 °C day/night temperatures under a 16 h/8 h light/dark photoperiod with 5500 Lx light intensity and relative humidity of 70–75%. The two-week-old seedlings with two fully expanded leaves from 10 single plants were then sampled, then frozen in liquid nitrogen rapidly. Finally, the treated samples were stored at −80 °C for protein extraction.

### Protein extraction

Wheat leaves were ground in liquid nitrogen, after which five volumes of ice-cold 10% (w/v) trichloroacetic acid (TCA) in acetone plus 0.07% (v/v) 2-mercaptoethanol were added and samples were held at −20 °C for 4 h. Next, the samples were centrifuged at 14,000 g for 30 min at 4 °C, after which the supernatants were discarded and the pellets were washed three times with ice-cold acetone. The pellets were vacuum-dried and resuspended in lysis buffer^22^ using a Votex for 2 h. The lysates were then sonicated at 80 W output with ten bursts of 10 s each, while being cooled on ice for 15 s between bursts, after which the suspension was centrifuged at 14,000 g for 40 min and 25 °C to remove insoluble materials. The cleared lysates were collected and quantified by a Bradford assay (BioRad, California) based on a bovine serum albumin (BSA) standard^23^.

### Tryptic digestion

A total of 10 mg of protein were added to dithiothreitol (DTT) (10 mM) and incubated for 2.5 h at 37 °C, after which they were cooled to room temperature, then alkylated with iodoacetamide (IAA) (50 mM) for 30 min at room temperature in the darkness. The DTT and IAA-treated proteins were diluted by adding 100 mM NH_4_HCO_3_ to urea to give a final concentration 1.5 M, after which they were digested with trypsin (Promega) at a trypsin-to-protein ratio of 1:50 overnight (18 h). Finally, the resulting peptides were collected as a filtrate. After centrifugation for 20 min at 10,000 g, the precipitates were removed and then the supernatants were desalted using Sep-Pak Classic C18 cartridges (Waters) followed by lyophilization.

### Enrichment of ubiquitin-remnant-containing peptides

Lyophilized peptides were dissolved in immunoaffinity purification (IAP) buffer (50 mM MOPS–NaOH, pH 7.2, 10 mM Na_2_HPO_4_, and 50 mM NaCl), then spun at 10,000 g at 4 °C for 10 min. di-Gly-Lys antibody beads were used (PTMScan ubiquitin remnant motif K-ε-GG kit, Cell Signaling Technology) and di-Gly-Lys-containing peptides were enriched as previously described^5^.

### Liquid chromatography (LC)-tandem mass spectroscopy (MS/MS) analysis

Enriched peptides were injected for LC–MS/MS analysis. The peptides were loaded onto a column (Thermo Scientific Acclaim PepMap 100, 100 μm × 2 cm, nanoViper C18) connected to an analytical column (Thermo Scientific Easy Column, 10 cm long, 75 μm inner diameter, 3 μm resin) in buffer A (0.1% formic acid), then separated with a linear gradient of buffer B (84% acetonitrile and 0.1% formic acid) at a flow rate of 300 nL/min. The gradient was as follows (0 min–220 min, 0–55% solvent B; 220 min–228 min, 55–100% solvent B; 228 min–240 min, solvent B at 100%). LC–MS/MS analysis was conducted on a Q Exactive mass spectrometer (Thermo Scientific). The mass spectrometer was operated in positive ion mode. MS data was acquired using a data-dependent top10 method dynamically choosing the most abundant precursor ions from the survey scan (300–1800m/z) for HCD fragmentation. Survey scans were acquired at a resolution of 70,000 at m/z 200 and resolution for HCD spectra was set to 17,500 at m/z 200. The normalized collision energy was 27 eV, and the underfill ratio, which specifies the minimum percentage of the target value likely to be reached at the maximum fill time, was defined as 0.1%. The mass spectrometry proteomics data have been deposited to the ProteomeXchange Consortium via the PRIDE^24^ partner repository with the dataset identifier PXD007243.

### Data analysis

The MS data were analyzed using the MaxQuant software (version 1.3.0.5). MS data were searched against the Uniprot_Triticum_aestivum_101036_20160308.fasta (released at March 08, 2016, 101036 sequences). During the database search, the modifications were set as follows: main search ppm: 6; missed cleavage: 4; MS/MS tolerance ppm: 20; De-Isotopic: TRUE; enzyme: trypsin; fixed modification: carbamidomethyl (C); variable modification: oxidation (M), acetyl (Protein N-term), GlyGly (K); decoy database pattern: reverse; iBAQ: TRUE; match between runs: 2 min; minimum peptide length: 7; false discovery rate (FDR) thresholds for proteins, peptides and modification sites: 0.01.

### Bioinformatics analysis

The Motif-X software was used to analyze the model of sequences with amino acids in specific positions of ubiquityl-15-mers (seven amino acids upstream and downstream of the ubiquitination site) in all of the protein sequences^16^. The *Arabidopsis thaliana* proteome was used as the background database, the setting parameters were: occurrences = 20, Bonferroni corrected *P*-value = 0.005 (motif-x significance = 0.00018), and the other parameters were set to the default values. Secondary structures of proteins were predicted by NetSurfP and *p* value was calculated as previously described^25^. The Gene Ontology (GO) annotation proteome was derived from http://www.ebi.ac.uk/GOA and the lysine ubiquitination (K^ub^) proteins were classified by GO annotation based on three categories: biological processes, cellular components and molecular functions. The Kyoto Encyclopedia of Genes and Genomes (KEGG) database was used to annotate protein pathway^26^. WoLF PSORT, a subcellular localization predication program, was used to predict subcellular localization^27^. In addition, protein–protein interaction information of the surveyed proteins was retrieved from STRING software (http://string-db.org/). The results were downloaded as the tsv format and were imported into Cytoscape (version 3.2.1) software^28^ (http://www.cytoscape.org/) to visualize and further analyze functional protein-protein interaction networks. However, since *Triticum aestivum* does not appear to be in the STRING database, thus its high sequence similarity species barley (*Hordeum vulgare* L.) was used for STRING (protein with the similar sequence probably have the same function). Furthermore, BLASTP was conducted to evaluate the conservation of lysine-ubiquitinated proteins among wheat, rice and *Arabidopsis thaliana* according to previous report^29^.

## Results

### Proteome-wide analysis of lysine-ubiquitinated sites in wheat

**Figure 1:**
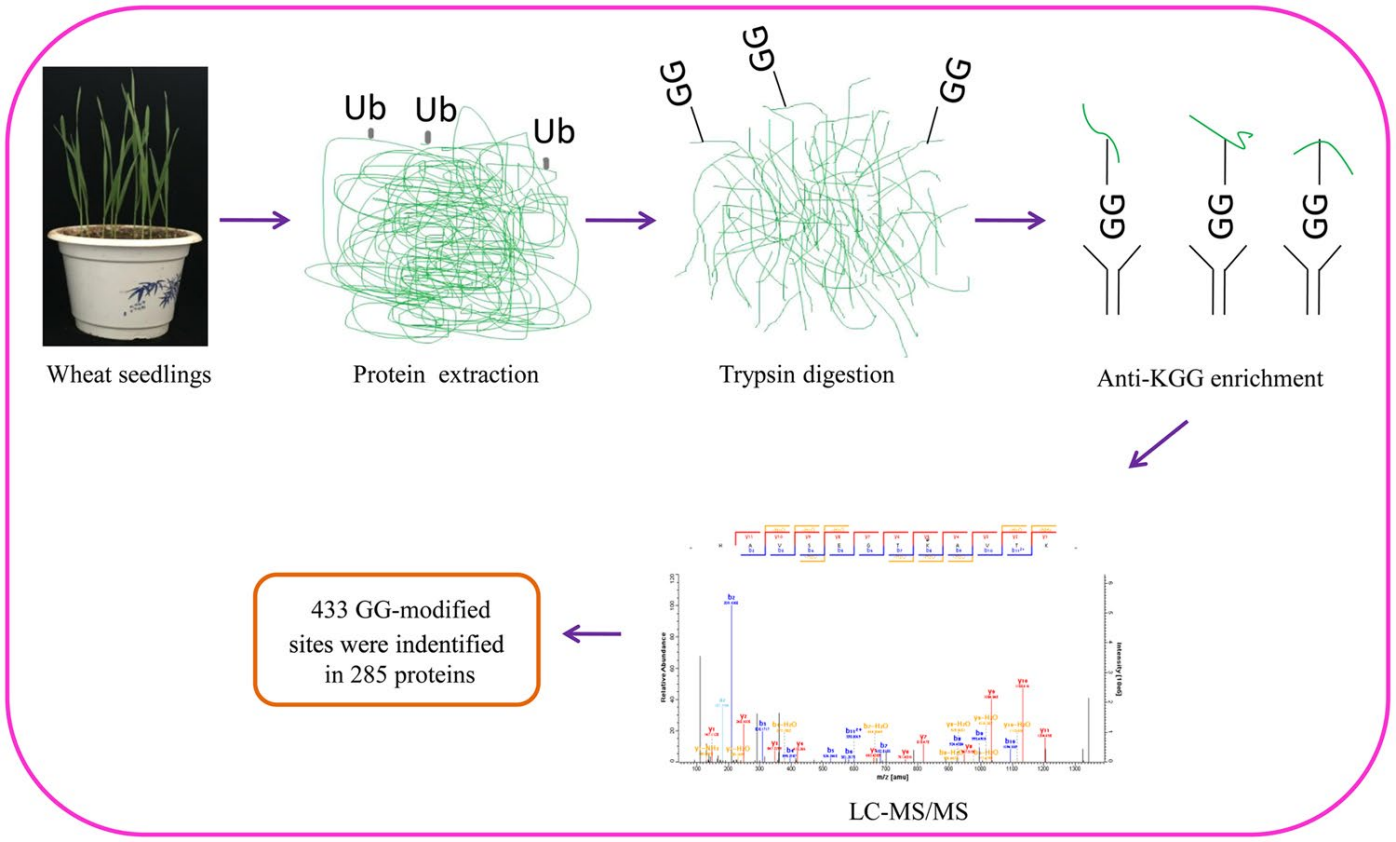
Overview of experimental procedures used in this study.

**Figure 2:**
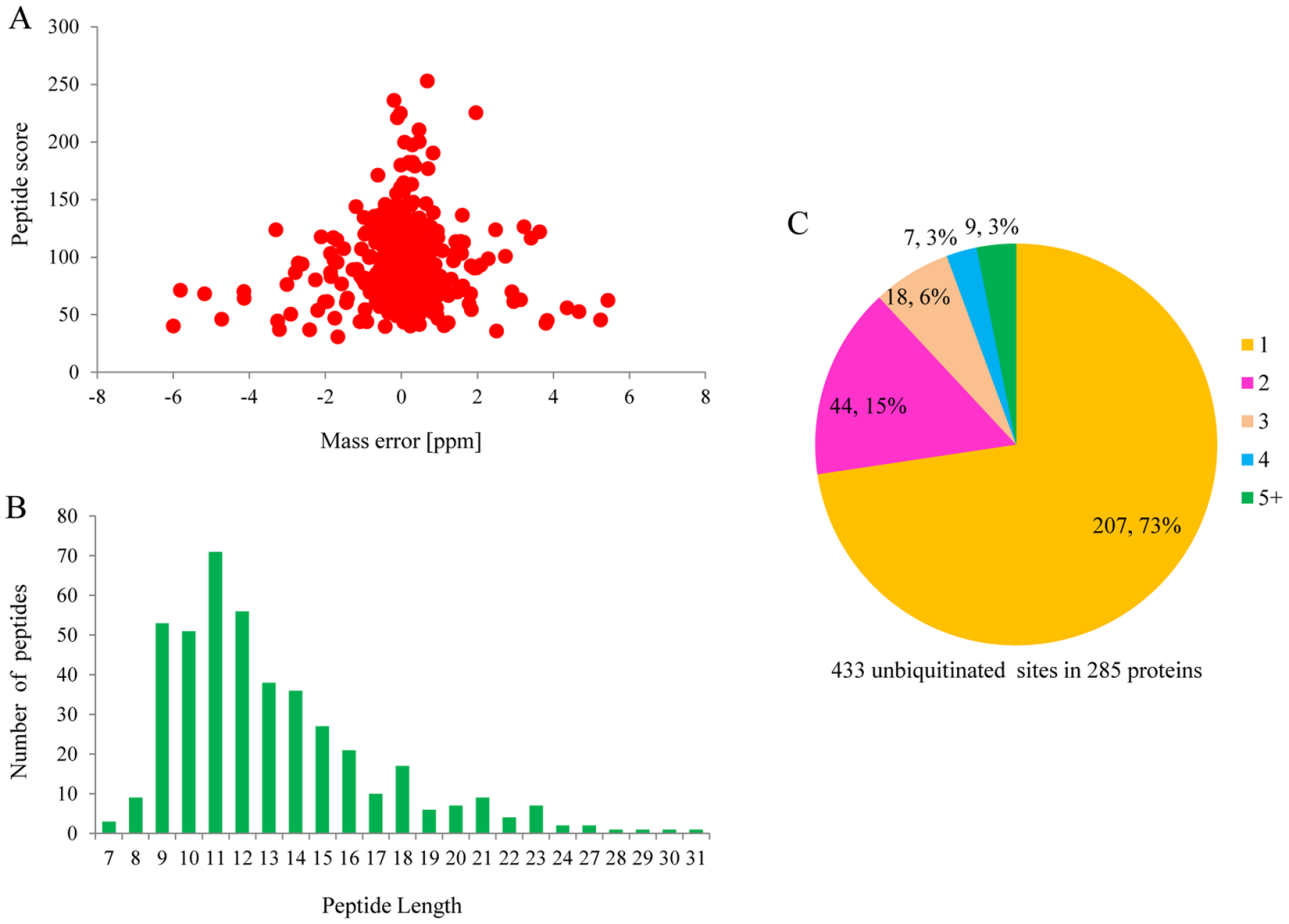
Proteome-wide identification of lysine ubiquitination sites in wheat. (**A**) Mass error distribution of all identified peptides. (**B**) Peptide length distribution. (**C**) Pie chart illustrating the number and percentage of lysine ubiquitination sites per protein.

An overview of experimental procedures used in this study is shown in Fig. 1. To identify lysine-ubiquitinated sites in wheat, proteins were extracted and digested into peptides with trypsin. Lysine-ubiquitinated peptides were then immune-enriched using a di-Gly-Lys-specific monoclonal antibody and analyzed by high-resolution LC-MS/MS. The mass errors of all identified peptides were checked and the results confirmed the high accuracy of the MS data (Fig. 2A). The length of the lysine-ubiquitinated peptides obtained was distributed between 7 and 31, which is in accordance with the property of tryptic peptides (Fig. 2B). Using this method, we identified 433 lysine-ubiquitinated sites in 285 unique proteins, which refer to 410 di-Gly-Lys-containing peptides (Additional file 1; Additional Fig. S1), and totals of 2505 non-modified peptides were identified (Additional file 2). To assess the distribution of ubiquitination sites in the lysine-ubiquitinated proteins of wheat, the numbers of identified modification sites per protein were calculated. The results indicated that 73% of proteins contained a single putative ubiquitination site, and the percentage of proteins with two, three, four and five or more modification sites were 13%, 5%, 4%, and 5%, respectively (Fig. 2C).

Trypsin proteolysis of proteins modified by ubiquitin, NEDD8, or ISG15 generates an identical di-Gly remnant on modified lysines, making it impossible to distinguish among these modifications by MS^15^. NEDD8-mediated modifications primarily target cullin subunits of cullin-RING E3 ubiquitin ligases in plants^30^, and the expression of ISG15 and its conjugation to lysines is relatively low in cells cultured under standard cell culture conditions^31^. Thus, both NEDDylation and ISGylation are very rare when compared to ubiquitination^32^. Consequently, a majority of cellular peptides containing the di-Gly remnant stem from ubiquitylated proteins. Therefore, in this study, we refer to all di-Gly modified lysines as “ubiquitylation sites,” even though a small fraction of these sites might be generated by modification of ISG15 or NEDD8.

### Motif analysis of lysine ubiquitination sites

**Figure 3:**
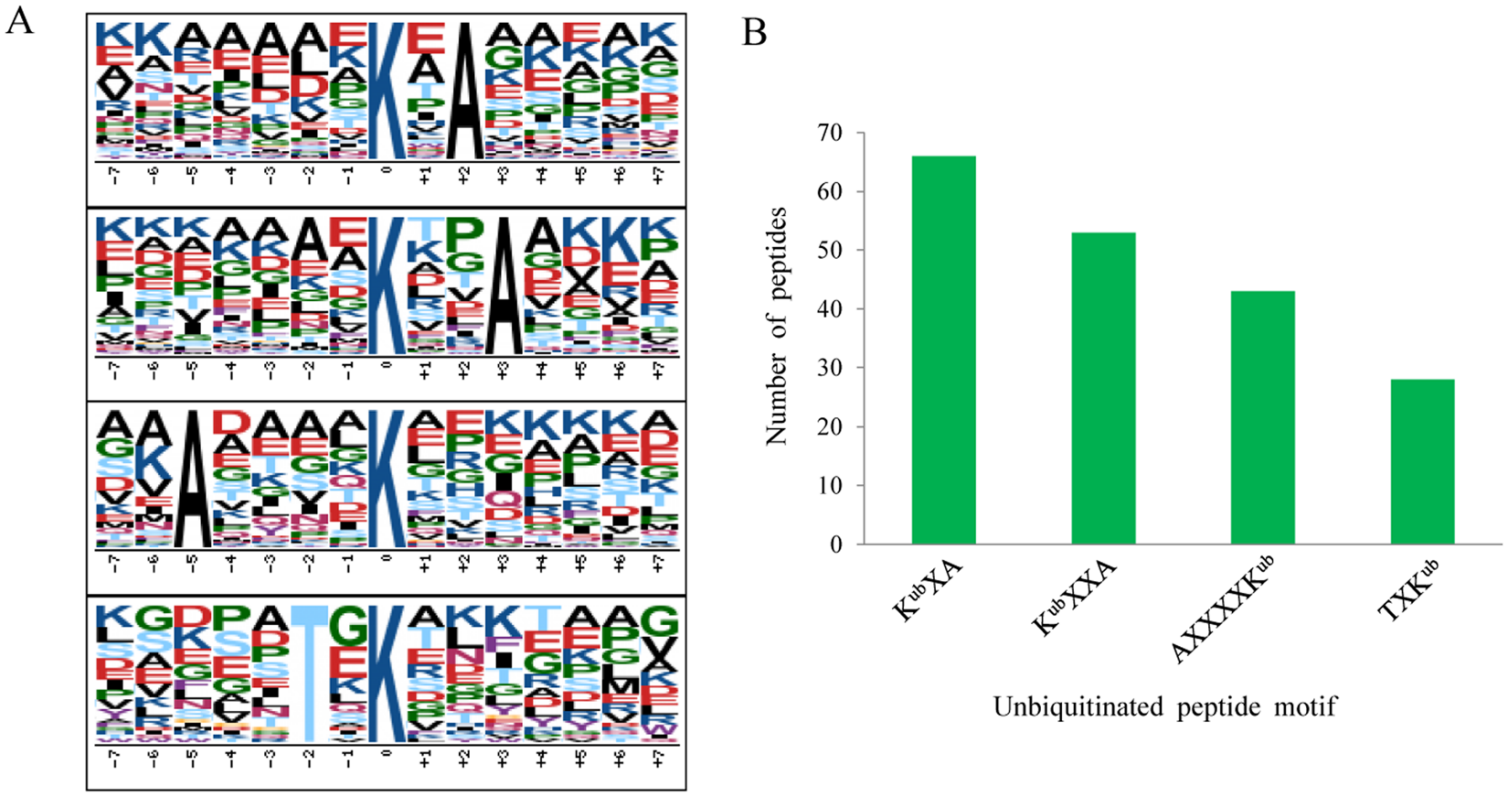
Ubiquitinated lysine motifs in wheat. (**A**) Ubiquitination motifs and the conservation of ubiquitination sites. The height of each letter corresponding to the frequency of the amino acid residue in that position. The central K stands for the ubiquitinated lysine. (**B**) The number of identified ubiquitinated peptides in each motif.

A previous study showed that conserved motifs might not exist in humans^1,5,12^, which is probably because ubiquitination sites lack the preference for specific amino acid residues at particular positions surrounding the ubiquitinated lysine in human cells. Or, this might be due to the use of an overly strict parameter setting during analysis by the Motif-X program. However, different organisms (e.g., mammals and plants) may have different sequence preferences in the ubiquitination sites. For instance, seven conserved motifs were identified in rice^16^. To further determine the nature of the ubiquitinated lysines in wheat, we analyzed the context of the amino acid sequence surrounding the ubiquitinated lysines using the motif-x program. Of the 410 di-Gly-Lys-containing peptides, 398 had seven or more amino acids N- and C terminally surrounding the ubiquitinated lysine. Substantial bias in the amino acid distribution was observed from position −7 to + 7 around the ubiquitinated lysines in the 398 peptides identified (Fig. 3A and Additional file 3). Four conserved sequences around the ubiquitination sites were found in the wheat ubiquitome; namely, K^ub^XA, K^ub^XXA, AXXXXK^ub^, and TXK^ub^ (K^ub^ indicates the ubiquitinated lysine, and X indicates any amino acid), which refer to 66, 53, 43, and 28 unique peptides, respectively, and these exhibit different abundances, together accounting for about 47.7% of the ubiquitinated peptides identified (Fig. 3B). Moreover, analysis of the ubiquitinated lysine motifs showed that enrichment of one residue with a hydrophobic side chain, alanine (A), was observed at the +2, +3 or −5 position. Another type of amino acid with hydrophillic side chain, threonine (T), was enriched in the −2 position (Fig. 3A). These results imply that amino acid residues with hydrophobic/hydrophillic side chains might be functionally important for ubiquitination on lysine residues of target proteins. It is noteworthy that K^ub^XXA, one of these four ubiquitylation motifs, was also observed in rice which possibly implies that lysine ubiquitylation is a conserved PTM among different species. These three novel motifs in wheat would potentially provide an ubiquitination binding loci for future studies.

**Figure 4:**
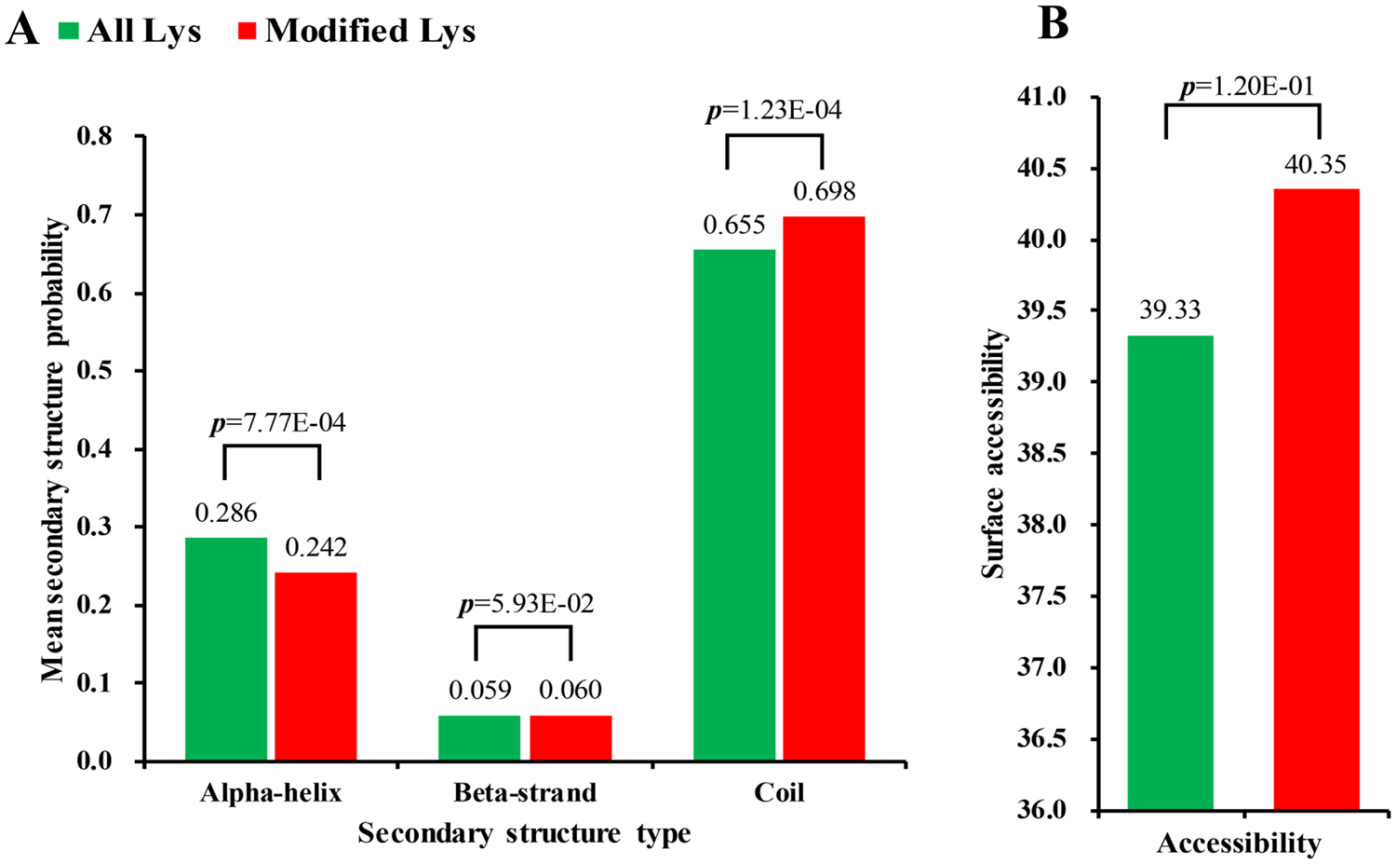
(**A**) Probabilities of lysine ubiquitination in different protein secondary structures (alpha-helix, beta-strand and coli). (**B**) Predicted surface accessibility of ubiquitination sites. All lysine sites were in green and ubiquitinated lysine sites were in red.

To elucidate the properties of ubiquitylation sites, the local secondary structures of protein sequences surrounding ubiquitylation sites was investigated (Fig. 4A). The results indicated that 30.2% of the ubiquitination sites were located at regions with ordered secondary structures (24.2% sites were located in the *α*-helix and 6% were in the *β*-strand), while 69.8% of the ubiquitination sites were located in the disordered structures of proteins (coil). Moreover, ubiquitylated lysines have a significantly different preference for secondary structures when compared with all lysines. In general, ubiquitylated lysines are found in ordered α-helix (*p* = 7.77e-04) and (*β*-strand *p* = 5.93e-02) regions more frequently, while they are less frequent in unstructured coil regions (*p* = 1.23e-04). In addition to ordered regions, we further evaluated our identified lysine ubiquitylation sites for solvent accessibility. The results showed that 40.35% of the ubiquitinated lysine sites were exposed to the protein surface when compared with 39.33% of all lysine residues (*p* = 1.20e-01) (Fig. 4B). Therefore, the surface properties of proteins are not likely to be changed by lysine ubiquitination.

### Functional classification of ubiquitinated proteins

**Figure 5:**
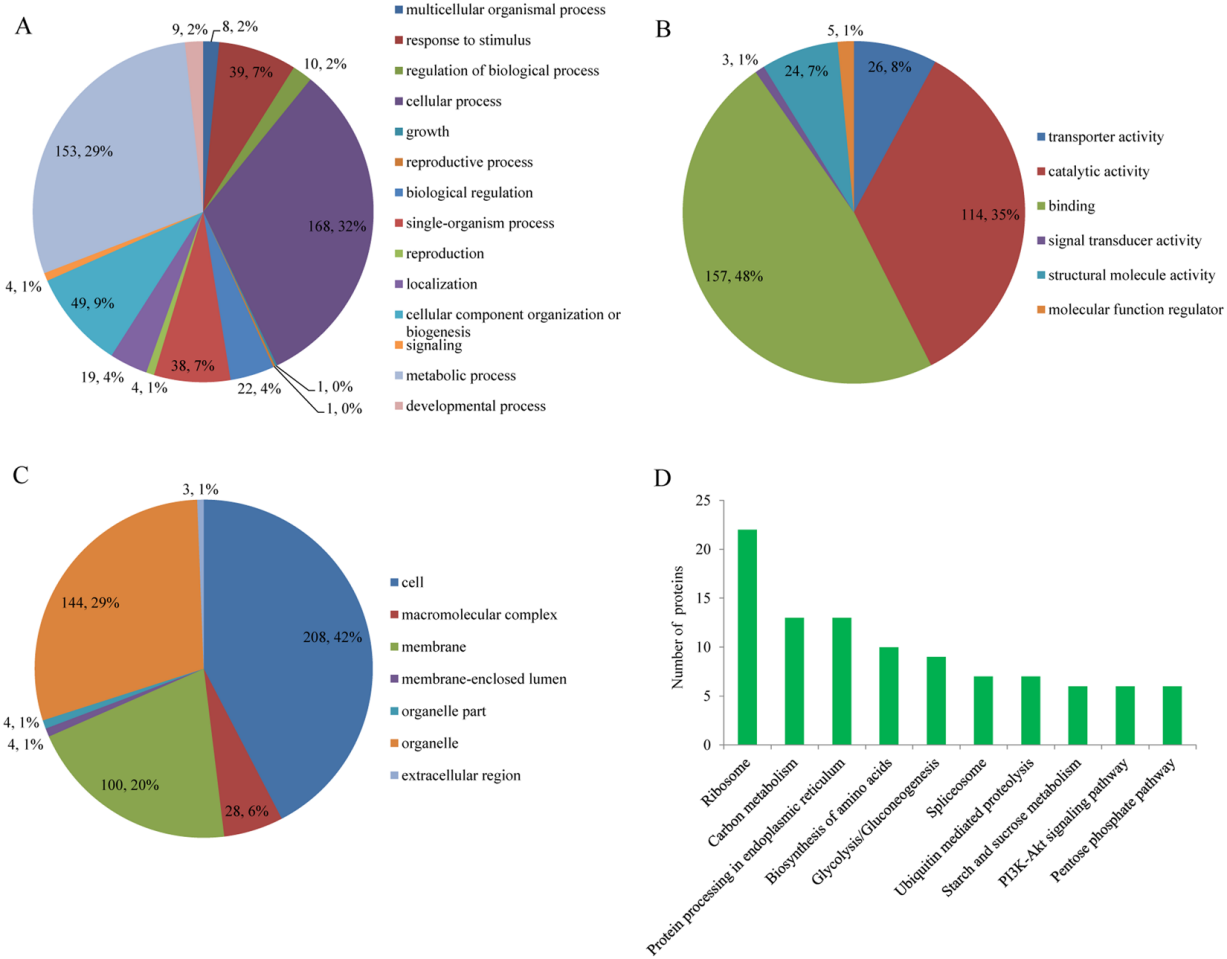
Pie charts of the distribution of ubiquitinated proteins based on their predicted molecular functions (**A**), biological processes (**B**), cellular components (**C**), and metabolic pathways (**D**).

To better understand the ubiquitome in wheat, GO functional classification of the identified ubiquitinated proteins was conducted based on the biological processes, molecular functions, and cellular components (Fig. 5A–C and Additional file 4). Among the 285 ubiquitinated proteins grouped by their biological processes, some were found to be related to cellular process (168, 32%) and metabolic process (153, 29%), while others were assigned to responses to stimuli (39, 7%), and single-organism processes (38, 7%) (Fig. 5A). Most ubiquitinated proteins in the molecular function classification were associated with binding activity (157, 48%) and catalytic activity (114, 35%) (Fig. 5B), suggesting that proteins may be involved in DNA transcription or protein interaction, and that enzymatic proteins are all subject to massive ubiquitination. Moreover, others were assigned to transporter activity (26, 8%) structural molecule activity (24, 7%), molecular function regulation (5, 1%), and signal transducer activity (3, 1%). Subcellular localization analysis revealed that 208 (42%) of the ubiquitinated proteins were located in cell, 144 (29%) in organelle, and 100 (20%) in the membrane (Fig. 5C). Furthermore, some proteins were predicted to be distributed in the macromolecular complex (28, 6%), membrane-enclosed lumen (4, 2%), organelle (4, 1%), and extracellular region (3, 1%) (Fig. 5C). The results of GO functional classification indicated that ubiquitinated proteins were involved in a broad range of biological processes and had various molecular functions in wheat. Below, we discuss the involvement of several biological processes in detail, specially notably enriched for proteins associated with proteasome composition, ribosome assembly/translation, membrane, carbohydrate metabolism, signal pathway, and photosynthesis (Table 1).

**Table 1:** Examples of pathways with ubiquitylation targets.

Accession no.^a^	Proteins	Accession no.^a^	Proteins
**26S proteasome**		Carbohydrate metabolism	
W5BUE8	26S proteasome non-atpase regulatory subunit 1	W5FGH0	UDP-glucose pyrophosphorylase
W5FXY6	26S proteasome non-atpase regulatory subunit 3	D8L9K9	Fructose-1,6-bisphosphatase, cytosolic,putative
W5DW28	26S proteasome non-atpase regulatory subunit 4	W5HCE5	Starch synthase I
A0A077RXS4	26S protease regulatory subunit 6 A homolog	W5HA05	Fructose-bisphosphate aldolase cytoplasmic isozyme
P31251	Ubiquitin-activating enzyme E1 2-like	C1J959	Fructose-bisphosphate aldolase cytoplasmic isozyme
W5B2T7	Ubiquitin-activating enzyme E1 3	W5HZ47	Fructose-bisphosphate aldolase isozyme 1
W5DFQ0	Ubiquitin-like 1-activating enzyme E1A	A0A096UTL2	Glyceraldehyde-3-phosphate dehydrogenase
W5FKD1	Ubiquitin-conjugating enzyme E2 variant 1C-like	A0A0C4BJ74	NADP-dependent glyceraldehyde-3-phosphate dehydrogenase
W5CBQ9	Ubiquitin-conjugating enzyme E2-17 kda	W5EAJ4	Glucose phosphomutase
W5D2H3	Ubiquitin-conjugating enzyme E2 27	W5B486	Xylulose kinase
W5BWF3	E3 ubiquitin- ligase HERC4	A0A077RSE3	Pyruvate kinase, cytosolic isozyme
W5AFH9	E3 ubiquitin- ligase MARCH11-like isoform ×2	W5FPI0	Enolase 2-like isoform ×2
W5H4P2	DNA damage-inducible protein 1	W5BG19	6-phosphogluconolactonase 2
W5EMB1	Cell division cycle 48	W5HDD8	Sucrose synthase
A0A068AZ53	WD-40 repeat-protein containing MSI4-like	W5HXV0	Fructokinase-2
**Ribosome assembly/translation**		W5B4C2	4-alpha-glucanotransferase DPE2-like
Q7XY23	40S ribosomal S3a-like	W5DRH6	Cellulose synthase
W5F4L4	40S ribosomal S29	A0A096UMJ7	Cellulose synthase-4
A0A077S4G0	40S ribosomal S2	**Stress/defence**	
W5I1R7	40S ribosomal S3	E9NVM6	Group 3 late embryogenesis abundant
U5HTD8	40S ribosomal S20	A0A096UP27	Wheat cold induced 16
W5G3X9	40S ribosomal S20	W5DJR4	Salt stress root RS1-like
W5AUH7	40S ribosomal S10	A0A077RZB4	Salt stress root RS1-like
Q5I7L0	60S ribosomal L18	P46524	Dehydrin WZY1-2
W5GGF8	60S ribosomal L13-2	P93608	Dehydrin WZY1-2
W5DEJ5	60S ribosomal L28-1-like	A7VL25	Group3 late embryogenesis abundant
W5CCH5	60S ribosomal L28-1-like	W5BA01	Early responsive to dehydration
W5F905	60S ribosomal L7a	A0A077RRB6	Glutaredoxin domain-containing cysteine-rich CG12206-like isoform 1
W5FIP7	60S ribosomal L4-1-like	P93612	Dehydrin 13
W5EW55	60S ribosomal L5-2	W5HHN0	Disease resistance RGA3
A0A077RTE5	60S ribosomal L9	W5G7J3	Temperature-induced lipocalin-1
W5C3Q8	60S ribosomal L9	W5FDB5	Disease resistance protein RGA2
A0A0C4BJE3	60S ribosomal L10a-1	A0A096ULF2	Probable glutathione S-transferase GSTU6-like
Q5I7L3	60S ribosomal L10a-1	W5DYG7	Superoxide dismutase [cu-zn] 2-like
W5HQA2	60S acidic ribosomal P1	W5AJ14	Thioredoxin H
Q5I7L1	The large subunit ribosomal proteins L13a	Q7FT21	Thioredoxin H
Q6EZA4	Large subunit ribosomal protein L40e	W5AMQ5	Thioredoxin H
W5A9E1	Ubiquitin-40S ribosomal protein S27a	W5EDY2	Glutathione peroxidase 4
**Chromatin-associated processes**		W5ETS3	Catalase 2
W5BYT5	Histone H1	**Singal pathway**	
O65795	Histone H1	W5BMZ7	Leucine-rich repeat transmembrane kinase 2
Q9XHL9	Histone H1	W5I1S6	Calcium-binding CML8
W5FG65	Histone H1	A0A096US65	Serine/threonine-protein kinase CTR1
O65794	Histone H1	W5GZ92	Seven transmembrane domain-containing tyrosine-protein kinase 1
W5A645	Histone H2A	W5BCP7	SAUR11 - auxin-responsive SAUR family member
W5DZG1	Histone H2A	W5E096	Rho guanine nucleotide exchange factor 6
W5HA06	Histone H2B.3	W5FB99	Phosphatase 2 A isoform 2 (PP2Ac-2)
W4ZWK0	Histone H2A	K4MQ41	Calcium-dependent kinase
W5A444	Histone H2B.2-like isoform 1	A0A0C4BJH4	14-3-3 GF14-D-like
A0A096UKH0	Histone H2A.1	L0GDQ5	14-3-3 B
W5GES9	Histone H2B.2	L0GED8	14-3-3 expressed
W5GGF1	Histone H2B.2	P04464	Calmodulin
W5H5H3	Histone H2B.2	**Transport**	
A0A096UKE9	H2B2_WHEAT ame: Full = histone	W5B354	Protein transport protein sec. 24-like
A0A096UTK7	Histone H2B.2	W5DXD2	Polyol transporter 4
W5HZ15	Histone H2B.2	A0A077RF08	Bidirectional sugar transporter SWEET1a-like
W5GH89	Histone H2A	W5FZC0	Sugar transport 14-like
W5FGP3	Histone H2A	A0A0E3IHU6	Inorganic phosphate transporter 1-4
Q43312	Histone H2A7	W4ZRP5	TOM1-like protein 2
W5BNS0	Histone H2A.4	W5HYJ9	Nitrate/chlorate transporter
A0A096USW9	H2B2 WHEAT ame: Full = histone	W5HN28	Lysine histidine transporter 1-like
W5DZU8	Histone -like isoform 1	C7C5T9	Sulfate transporter -like
W5EA78	Histone -like isoform 1	W5H0U1	TOM1-like protein 2
W5E0R4	Histone -like isoform 1	W5E117	Low affinity sulfate transporter 3-like
W4ZTZ4	Histone -like isoform 1	W5D0X5	Amino acid permease
A0A0C4BKM5	Histone H2A variant 1	**Photosynthesis**	
W5F1A1	Histone H2A variant 3	P83970	Plasma membrane H+ -atpase
W5A7J0	Histone H3	Q2L9B8	Vacuolar ATP synthase subunit E
A0A096USG9	Histone H4	W5APB1	Oxygen-evolving enhancer chloroplastic
**Protein folding**		A0A077RAG2	Cytochrome b5
A7LM55	Cyclophilin 1	P69415	Photosystem I subunit VII
W5A8B5	Heat shock 70 kda 4L	W5E7J2	Psbp chloroplastic-like protein
F4Y592	Heat shock protein 90	W5DWY0	Psbp chloroplastic-like protein
F4Y595	Heat shock protein 90	W5BG62	Ribulose 1,5-bisphosphate carboxylase/oxygenase large subunit
W5FEE5	Heat shock cognate 70 kda protein 1-like isoform 1	W5HZ47	Fructose-bisphosphate aldolase isozyme 1
W5BDM2	Heat shock cognate 70 kda protein 1	C1J959	Fructose-bisphosphate aldolase cytoplasmic isozyme
W5DZG0	Heat shock cognate 70 kda protein	W5HA05	Fructose-bisphosphate aldolase cytoplasmic isozyme
Q9SAU8	HSP70	D8L9K9	Fructose-1,6-bisphosphatase, cytosolic,putative
F8RP11	HOP	A0A096UTL2	Glyceraldehyde-3-phosphate dehydrogenase
**Cytoskeleton building**		**Mitochondrial fission**	
W5EGW3	Kinesin-4	W4ZR59	Mitochondria fission 1 protein
W5DRH6	Cellulose synthase	**Cell cycle progression**	
A0A096UMJ7	Cellulose synthase-4	A0MA43	Ran-binding protein
W5ETI1	Actin-1	**mRNA Processing**	
A0A0A7NVN8	Actin depolymerizing factor	W5ADX0	Pre-mRNA-processing factor 19-2-like protein
W5BLI5	Mixed-linked glucan synthase 3	**Sulfur metabolism**	
**Fatty acid metabolism**		W5FLB0	Cysteine synthase
A0A077RUB2	Phospholipase A1-II 5-like	**Apoptosis**	
W5DS68	Lipoxygenase 3	W5GE21	Apoptosis-inducing factor-like protein A
W5FLT9	Long-chain-fatty-acid–coa ligase 4	**Nitrogen metabolism**	
A0A096UUN4	Delta-12 oleate desaturase	Q45NB6	Glutamine synthetase isoform GS1b
W5E0C4	3-ketoacyl-coa synthase 6	**RNA edit**	
**Membrane-associated proteins**		W5CBR8	Pentatricopeptide repeat-containing mitochondrial-like (PPR)
A0A077S2A9	Patellin-5-like	**Flavonoid metabolism**	
W5ASB4	Aquaporin 8	W5BZL1	Chalcone synthase
A7J2I2	Plasma membrane intrinsic protein	**DNA repair**	
A7J2I3	Plasma membrane intrinsic protein	W5FH53	DNA repair RAD23
Q9M7C4	Plasma membrane intrinsic protein 1	W5FZL2	DNA repair RAD23
W5FVE5	Probable aquaporin PIP2-7-like	W5HG98	DNA repair RAD23

^a^Accession no.: accession number in Uniprot database. See Additional file 4 for the complete list (according to the GO annotation).

**Figure 6:**
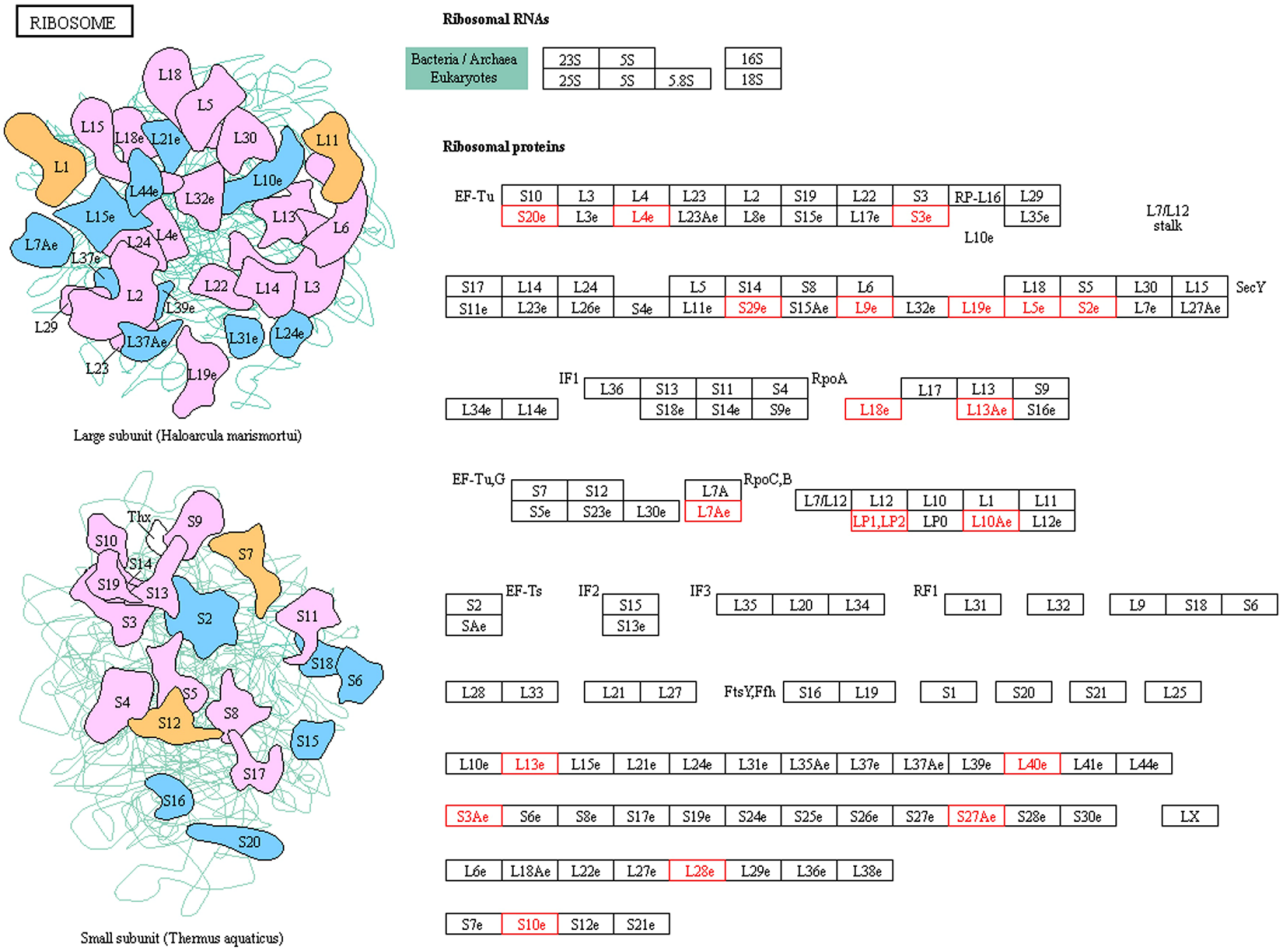
Ubiquitinated subunits of the 40S and 60S ribosome complexes in wheat. Ubiquitinated subunits are highlighted in red. Source: Kanehisa *et al*.^26^.

**Figure 7:**
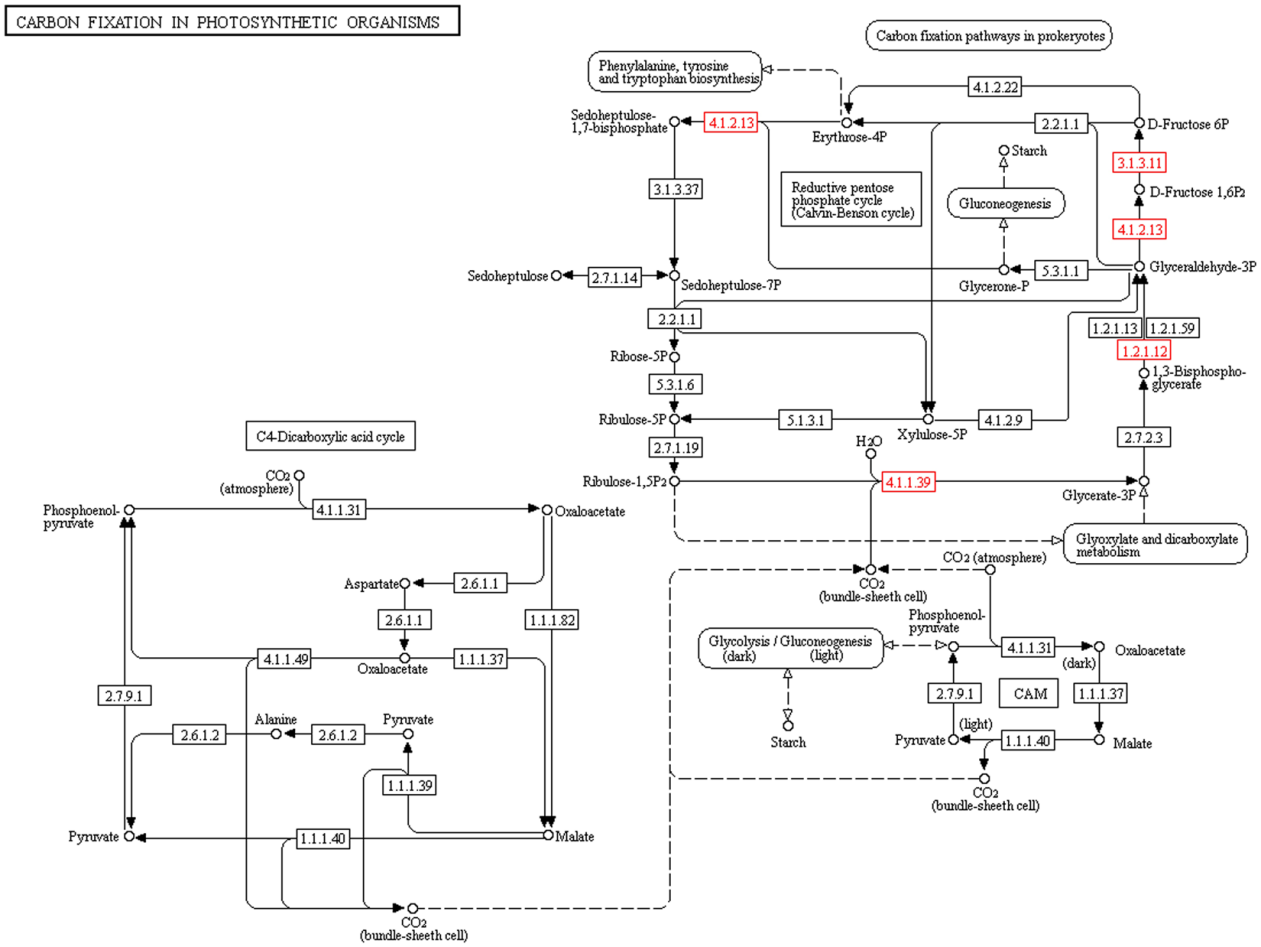
Ubiquitinated proteins in representative metabolic pathways in terms of carbon fixation in photosynthetic organisms in wheat. Ubiquitinated proteins are highlighted in red. W5HZ47, C1J959 and W5HA05 corresponds to 4.1.2.13, W5BG62 corresponds to 4.1.1.39, D8L9K9 corresponds to 3.1.3.11, and A0A096UTL2 corresponds to 1.2.1.12. Source: Kanehisa *et al*.^26^.

To gain insight into the ubiquitination mediated metabolic processes in wheat, we conducted KEGG pathway analysis. The results indicated that 285 ubiquitinated proteins were involved in 173 metabolic pathways (Fig. 5D and Additional file 5), primarily those associated with ribosome (22), carbon metabolism (13), protein processing in endoplasmic reticulum (13), biosynthesis of amino acids (10), and glycolysis/gluconeogenesis (9) (top 5, plant category). For example, in this study, a large set of 22 subunits of the 40S and 60S ribosome complexes ubiquitylated in wheat were identified (Fig. 6). During photosynthesis, the Calvin cycle converts carbon dioxide and other compounds into glucose. Several metabolic enzymes involved in the Calvin cycle were found to be ubiquitinated in wheat, including three isozymes of fructose-bisphosphate aldolase (W5HZ47, C1J959 and W5HA05), one ribulose 1,5-bisphosphate carboxylase/oxygenase large subunit (Rubisco) (W5BG62), one cytosolic putative fructose-1,6-bisphosphatase (D8L9K9), and one glyceraldehyde-3-phosphate dehydrogenase (A0A096UTL2) (Fig. 7).

### Protein interaction network of ubiquitinated proteins in wheat

**Figure 8:**
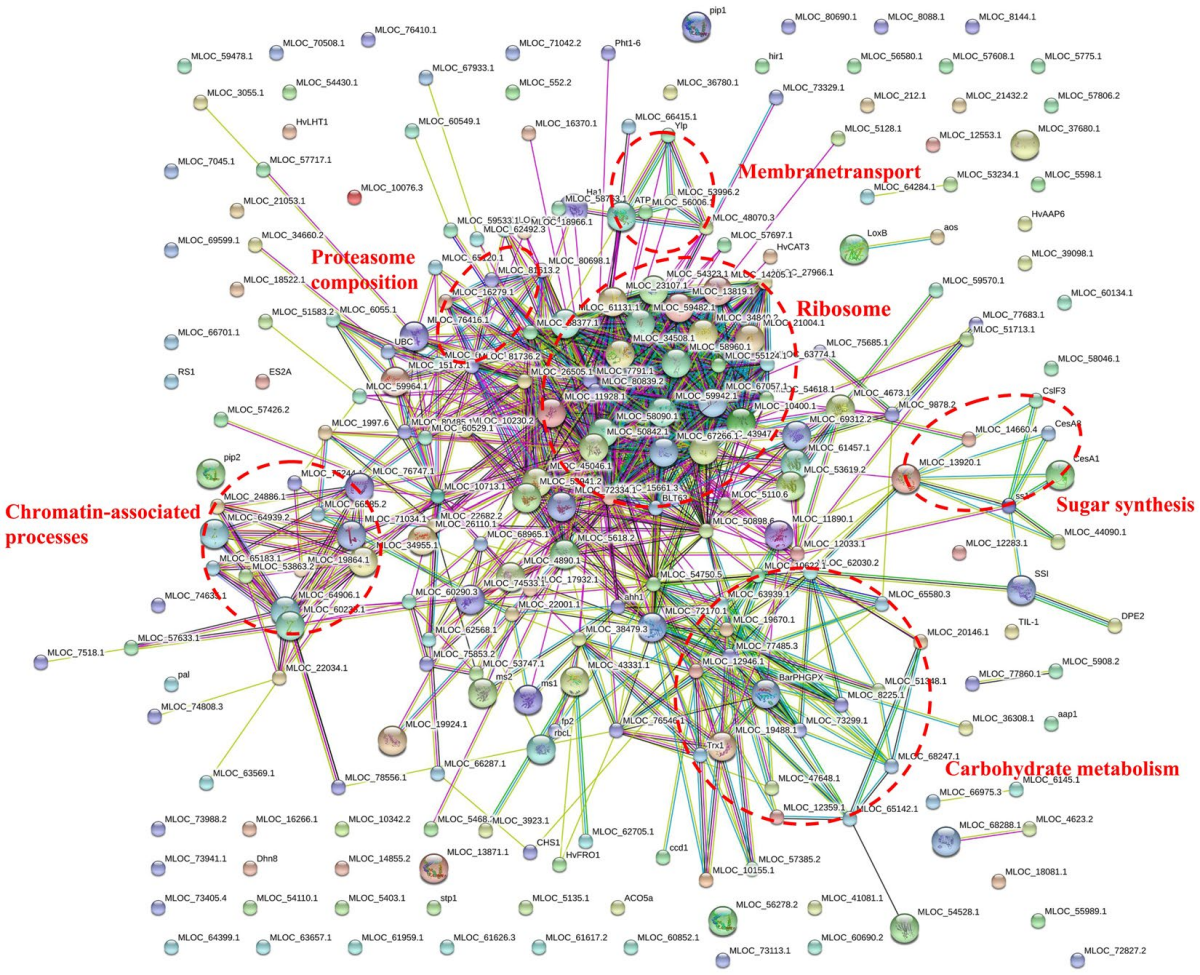
Interaction networks of the ubiquitinated proteins in wheat using String software. Different colored lines represent types of evidence for association: green, neighborhood evidence; red, fusion evidence; purple, experimental evidence; light blue, database evidence; black, coexpression evidence; blue, co-occurrence evidence; and yellow, text-mining evidence.

To further understand cellular processes regulated by ubiquitination in wheat, the protein interaction network was established (Fig. 8 and Additional file 6). Ubiquitylated proteins were grouped using associated GO biological process terms. The results showed that a total of 220 ubiquitinated proteins were mapped to the protein interaction database, which presents how ubiquitylated proteins perform diverse functions in wheat. This network identified a complex interconnected web with a number of ubiquitylated proteins present at key hubs, with subnetworks involved in carbohydrate metabolism, sugar synthesis, ribosome assembly/translation, 26S proteasome composition, membrane transport (aquaporins and H+ -ATPases), and chromatin-associated processes being especially enriched (Fig. 8). For example, among the clusters in the network, Cluster I-II consist of proteins involved in ribosome-associated and carbohydrate metabolism, while Cluster III refers to various ubiquitylated histones that may play important roles in regulation of chromatin-associated processes. The clusters of these three pathways all displayed dense protein interaction networks. The physiological interactions among these proteins may lead to their cooperation and coordination in wheat.

### Conservation analysis of the ubiquitinated proteins

**Figure 9:**
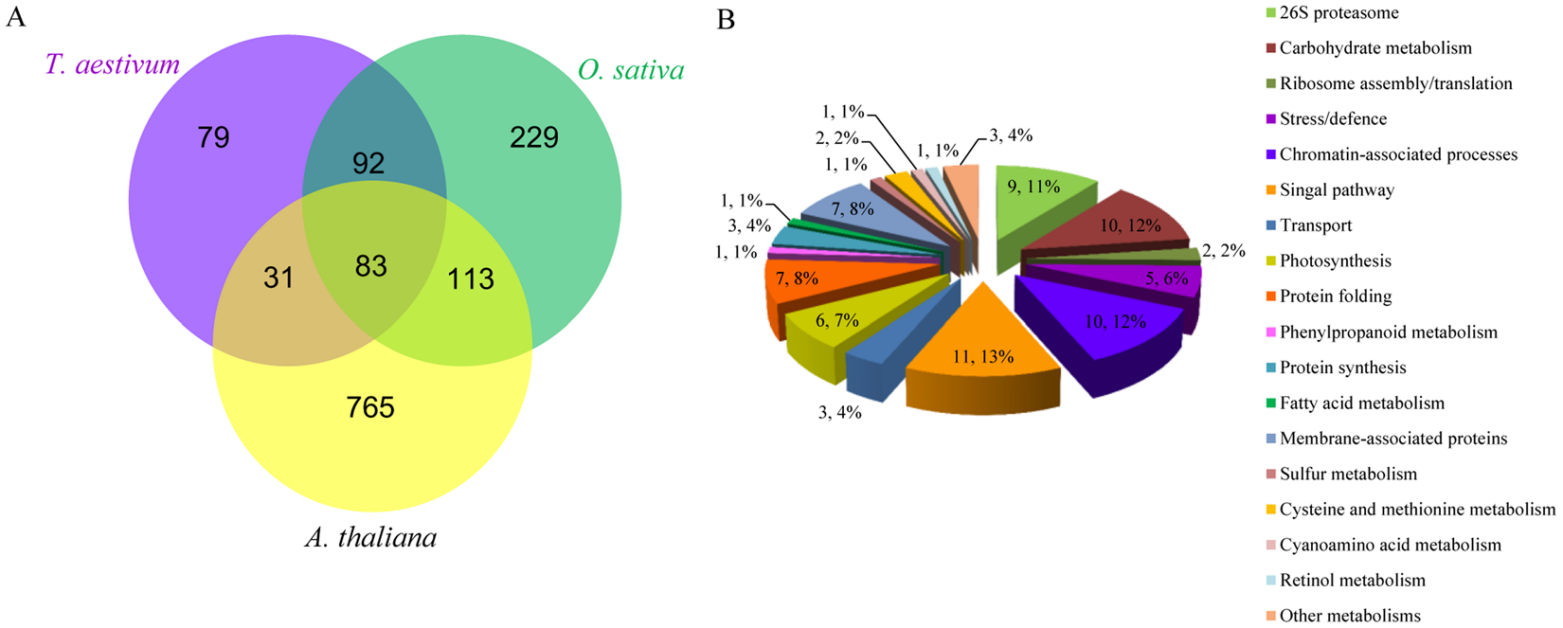
Conservation analysis of the ubiquitinated proteins in wheat. (**A**) All the identified ubiquitinated proteins in wheat compared with *Oryza sativa*, *Arabidopsis thaliana*. (**B**) Functional classification of the common ubiquitinated proteins identified in wheat, *Oryza sativa*, and *Arabidopsis thaliana*.

To reveal the commonality and specificity of lysine ubiquitination between wheat and other species, we used the sequences of the identified proteins to perform a BLAST search and estimated the degree of conservation of ubiquitinated proteins among wheat, rice, and *Arabidopsis thaliana*. The parameters were set as follows: E-value <1 × 10^−10^, score ≥80, and identity ≥30%. As shown in Fig. 9A, 206 (72.3%) of the identified ubiquitinated proteins in wheat had orthologous proteins in the other two species. A total of 83 ubiquitinated proteins were found in all three species (Additional file 7; Fig. 9A). Further analysis demonstrated that most of these orthologous proteins were involved in signal pathway, carbon metabolism, chromatin-associated processes, 26S proteasome, and protein folding (Additional file 8; Fig. 9B). Among the 285 identified ubiquitinated proteins in wheat, 31 had conserved orthologs with an average identity of 80.9% in *Arabidopsis thaliana* (Additional file 7; Fig. 9A), which involved in ribosomal proteins, 26S proteasome subunits and cytoskeleton building proteins. However, 92 had highly conserved orthologs with an average identity of 84.6% in rice (Additional file 7; Fig. 9A), and most of these proteins participated in chromatin-associated processes and stress/defence. This indicated that ubiquitination is conserved in different species and plays important roles in signal pathway and chromatin-associated processes. In addition, 79, 229, and 765 ubiquitinated proteins were only found in wheat, rice, and *Arabidopsis thaliana*, respectively (Additional file 7). For instance, ubiquitinated proteins associated with mitochondrial fission, apoptosis and cell cycle progression were only identified in wheat (Table 1). These results imply that lysine ubiquitination plays both common and specific roles in different plant species.

## Discussion

### Strategy for ubiquitome in wheat

Protein conjugation with ubiquitin, known as ubiquitination, is a well conserved post-translational modification in eukaryotic organisms that plays important roles in many cellular processes. Until recently, the ubiquitome was only reported in two plant species, *Arabidopsis* and rice (Oryza sativa)^1,16–21^. Obviously, the proteome-wide ubiquitination data are lacking for many other plant species. In this study, we conducted a proteomics study of lysine ubiquitination in common wheat, which is one of the most important crops in the world. Through combining the highly specific enrichment of lysine-ubiquitinated peptides with highly sensitive LC-MS/MS, 433 lysine modification sites were identified on 285 proteins.

Previous studies mainly focused on either the use of overexpression of epitope-tagged ubiquitin or ubiquitin binding domains/antibodies to capture ubiquitylated proteins for identification by MS^11–13,33,34^. However, the low occupancy of ubiquitylation challenges detection of endogenously modified proteins in the absence of overexpression of either ubiquitin or substrate^15^. A highly robust and streamlined proteomic method to map endogenous putative ubiquitylation sites precisely was utilized in this study. The advantages over previously described MS-based methods for ubiquitylation sites identification are as follows: (1) it can identify ubiquitylation sites in any tissue or organism; (2) it analyzes endogenous ubiquitylation sites in-depth on the proteome-wide level; (3) it is highly efficient at enriching the ubiquitylated peptides, enabling detection of low abundant modification sites; (4) it only requires a single-step affinity enrichment of modified peptides; (5) it is fully compatible with proteome-wide site-specific quantification of ubiquitylation.

### Ubiquitylation regulates diverse biological processes

The ubiquitinated proteins identified in this study belong to diverse functional groups based on their GO annotations and are localized to multiple cellular compartments, indicating that lysine ubiquitination plays important roles in regulating many cellular processes in wheat. Protein interaction network analysis demonstrated that a broad range of interactions are modulated by protein ubiquitination. Furthermore, the conservation analysis of the ubiquitinated proteins between wheat and other species imply that lysine ubiquitination displays both commonality and specificity in different plant species. This study provides the first comprehensive view of the ubiquitome in wheat.

In the 26S proteasome, four core subunits (W5BUE8, W5FXY6, W5DW28, A0A077RXS4) were found to be ubiquitylated. Additionally, other UPS components including the ubiquitin-like 1-activating enzyme E1A (W5DFQ0), ubiquitin-conjugating enzyme E2 27 (W5D2H3), E3 ubiquitin-ligase MARCH11-like isoform X2 (W5AFH9), DNA damage-inducible protein 1 (Ddi1) (W5H4P2), cell division cycle 48 (CDC48) (W5EMB1), and WD-40 repeat-protein O (A0A068AZ53) were notably enriched in our wheat ubiquitylome catalog, similar to the results of studies investigating other eukaryotes^5,7,12,15^. One example is CDC48, which plays a central role in the UPS-dependent turnover of misfolded ER-resident proteins after their retrograde transport back to the cytosol^35^ as reported to be ubiquitylated in yeast^11,36^. Ubiquitinated proteins are either directly recognized by the proteasomal Ub receptors or first bound by the so-called shuttle proteins, which then drive the ubiquitinated proteins to the 26S proteasome for degradation^37–39^. Ddi1 belongs to a family of shuttle proteins targeting polyubiquitinated substrates for proteasomal degradation^40^. One Ddi1 identified in this study was also found in rice ubiquitome^16^. However, E3 ubiquitin-ligase MARCH11-like isoform ×2 and WD-40 repeat-protein O were first identified in wheat. These findings suggest that such autoubiquitylation has regulatory consequences or might simply reflect collateral damage caused by their proximity to the Ub-transfer machinery.

Evidence of ribosomal subunits being ubiquitylated has previously been presented for *Arabidopsis* ^17,20^ and other eukaryotes^5,7,11,15,41^. This modification could represent a regulatory step during ribosome assembly and/or translation, or implicate the UPS in the removal of improperly folded subunits or entire ribosomes when they become non-functional or less important upon cell starvation^42^. In this study, a large set of ubiquitylated subunits of the 40S and 60S ribosome complexes in wheat were identified (Fig. 6), implying that ubiquitination is likely to be an important regulatory mechanism for ribosomal proteins. Ubiquitylation plays important roles in protein trafficking and membrane protein turnover^43,44^. In this study, the presence of H+ -ATPase, aquaporin, intrinsic protein, and patellin in the ubiquitylome catalog suggest a vital role for Ub in controlling plasma membrane protein activity and/or turnover in wheat, likely via an endocytosis pathway that extracts the receptors from the membrane and delivers them to the vacuole for turnover^17^. A previous study indicated the expression of a fusion between H+ -ATPase AHA1 and Ub in *Arabidopsis* was sufficient to induce its endocytosis and sorting into the vacuolar lumen^45^. Furthermore, a previous study in other eukaryotes indicated that histones or isoforms were modified by PTMs, such as phosphorylation, acetylation and methylation, and ubiquitination, which are known to play important roles in the regulation of chromatin-associated processes^46^. We identified some ubiquitylation sites on various histones, including five major histones (H1, H2A, H2B, H3, and H4), several isoforms of histone, and two histone H2A variants. Generally, these results demonstrate the importance of ubiquitylation and the UPS in the catabolism and trafficking of membrane-associated proteins.

Carbohydrate metabolism regulates sugar synthesis and transformation as well as carbon partitioning. The process is a highly critical metabolic pathway in plants^47^. In this study, a large number of ubiquitylation sites were observed on carbohydrate metabolism-related enzymes. Notable examples in carbohydrate metabolism include those that play important roles in glycolysis, such as glyceraldehyde-3-phosphate dehydrogenase (A0A096UTL2, A0A0C4BJ74), enolase 2-like isoform ×2 (W5FPI0), the cytosolic isozymes of pyruvate kinase (A0A077RSE3) and fructose-bisphosphate aldolase (C1J959, W5HA05, W5HZ47); and those that occupy key cytosolic steps in sugar formation and metabolism, such as starch synthase I (W5HCE5), glucose phosphomutase (W5EAJ4),sucrose synthase (W5HDD8), fructokinase-2 (W5HXV0), 4-alpha-glucanotransferase DPE2-like (W5B4C2) and UDP-glucose pyrophosphorylase (W5FGH0); as well as those that is vital in pentose phosphate pathway (PPP), such as xylulose kinase (W5B486), and 6-phosphogluconolactonase 2 (W5BG1). In support of our findings, the majority of the enzymes found to be related carbohydrate metabolism in this study were lysine ubiquitylated in *Arabidopsis* and rice^48,49^. These findings highlight the notion that lysine ubiquitylation plays a key regulatory role in the process of carbohydrate metabolism.

Diverse cell surface receptors and intermediate signaling components in this study were found to be ubiquitylated. In plants, receptor tyrosine kinases (RLKs) mediate many signaling messages at the cell surface and act as key regulators during developmental processes^50^. A previous study indicated that ubiquitylation of RTKs controls the amplitude and duration of receptor signaling^51^. We also mapped several ubiquitylation sites on tyrosine-protein kinase (RTKs) (W5GZ92) and leucine-rich repeat transmembrane kinase 2 (LRR-RLKs) (W5BMZ7). Other cell surface receptors and intermediate signaling components, such as 14–3–3, which function as homodimers or heterodimers and bind a large number of differentially phosphorylated substrates to regulate a wide array of cellular signaling and physiological processes, were ubiquitylated. These include calmodulin, an intracellular target of the secondary messenger Ca^2+^ that acts as part of a calcium signal transduction pathway by modifying its interactions with various target proteins such as kinases or phosphatases^52,53^. The serine/threonine-protein kinase CTR1, Rho guanine nucleotide exchange factor 6, phosphatase 2A isoform 2 (PP2Ac-2), calcium-dependent kinase (CDPKs), and auxin-responsive SAUR family members were also ubiquitylated. These proteins are involved in a variety of signaling pathways such as the calcium signaling pathway, PI3K-Akt signaling pathway, ethylene signal transduction, and abscisic acid, auxin hormone signaling. The majority of cell surface receptors and intermediate signaling components are also present in *Arabidopsis thaliana* ^17,54^ and human ubiquitome^15^; however, auxin-responsive SAUR family member and Rho guanine nucleotide exchange factor 6 have not been reported previously. The identification of ubiquitylation sites on cell surface receptors and major components of the signal pathways indicates that ubiquitylation plays a broad regulatory role in these signaling pathways in wheat. These findings also suggest that Ub addition controls the crosstalk between pathways.

Proteins involved in photosynthesis were identified in the wheat ubiquitome. The conversion of light energy to chemical energy in photosynthesis involves electron transfer and ATP synthesis. Driven by light, photosystem II (PSII) catalyzes electron transfer from water to plastoquinone. PsbP is a thylakoid luminal subunit of photosystem II (PSII) that plays an important role in maintaining photosynthetic electron transfer^54^. Unlike PsbP proteins, PsbP-like protein has been implicated in the assembly, stability, and/or repair of PSII complexes or subunits^55^. We mapped ubiquitylation sites on 2 psbP-like proteins (W5E7J2 and W5DWY0). Additionally, other components important for electron transfer such as cytochrome b5 (Cyt b5) (A0A077RAG2), oxygen-evolving enhancer chloroplastic (W5APB1), and vacuolar ATP synthase subunit E (P83970) were identified as ubiquitylated proteins in wheat. These findings demonstrate that lysine ubiquitylation plays a key regulatory role in the process of photosynthesis.

Wheat provides a large amount of starch ever year that is synthesized from one product of photosynthesis, glucose^56^. During photosynthesis, the Calvin cycle converts carbon dioxide and other compounds into glucose. Six metabolic enzymes in the Calvin cycle were found to be ubiquitinated in wheat (Fig. 7). Similar results were also found in the rice ubiquitome^16^. Rubisco is a major protein in the stroma of chloroplasts that fixes carbon dioxide as the first step of the Calvin cycle via catalyzing the carboxylation of ribulose-1,5-bisphosphate^57^. These studies suggest that, in plants, lysine ubiquitylation participates in regulation of the activity of enzymes in the Calvin cycle.

Ubiquitylated proteins were found to be participating in nuclear as well as nonnuclear processes, including DNA repair, apoptosis, and protein folding. For example, three DNA repair RAD23 (W5FH53, W5FZL2, and W5HG98) were modified by ubiquitylation. RAD23 is an evolutionarily conserved protein that is important for nucleotide excision repair^58^. The stabilization of DNA repair and stress factors could represent an important biological function of RAD23^59^. A previous study also indicated that RAD23 links DNA repair to the ubiquitin/proteasome pathway^58^. Moreover, one apoptosis-inducing factor-like protein A (W5GE21) and two heat shock proteins (HSP 90 and HSP70) were found to be ubiquitylated. Particularly notable, we found numerous ubiquitylated proteins involved in stress/defense, transport, nitrate metabolism, sulfur metabolism, fatty acid metabolism, cell wall metabolism, flavonoid metabolism, mitochondrial fission, cell cycle progression, mRNA processing (Table 1) and other metabolic processes, suggesting that these processes are heavily influenced by Ub addition. Besides, some ubiquitylated proteins were identified for the first time in this study.

Overall, this study provides the first extensive data on lysine ubiquitination in common wheat, and enhances the concept that ubiquitination mediates diverse cellular processes, especially with respect to proteasome composition, ribosome assembly/translation, carbohydrate metabolism, signal transduction, and photosynthesis. Our findings broaden the extent of physiological processes regulated by lysine ubiquitination and will serve as a valuable reference for the functional analysis of lysine ubiquitination in wheat and other plants.

## Electronic supplementary material

Additional Figure S1

Additional file 1

Additional file 2

Additional file 3

Additional file 4

Additional file 5

Additional file 6

Additional file 7

Additional file 8
