# Supplementary material for: Comprehensive profiling of lysine ubiquitome reveals diverse functions of lysine ubiquitination in common wheat
Source: Sci Rep. 2017 Oct 19;7:13601. doi: 10.1038/s41598-017-13992-y (PMC5648756; doi:10.1038/s41598-017-13992-y)
Supplement: Supplementary file 1 — Additional Figure S1 [file 41598_2017_13992_MOESM1_ESM.doc]

**Supplementary information**

**Comprehensive profiling of lysine** **ubiquitome reveals diverse functions of lysine** **ubiquitination in common wheat**

Ning Zhang, Lingran Zhang, Chaonan Shi, Qiuzhen Tian, Guoguo Lv, Ying Wang, Dangqun Cui, Feng Chen*

Agronomy College/National Key Laboratory of Wheat and Maize Crop Science/Collaborative Innovation Center of Henan Grain Crops, Henan Agricultural University, Zhengzhou 450002, China

^*^ Corresponding authors: F. Chen (chf0088@163.com)

Agronomy College, Henan Agricultural University, 95 Wenhua Road, Zhengzhou 450002, P. R. China

Phone: +86-371-63558537

**Additional Figure S1**. Examples of raw mass spectra in wheat ubiquitome.

**W5FXY6**


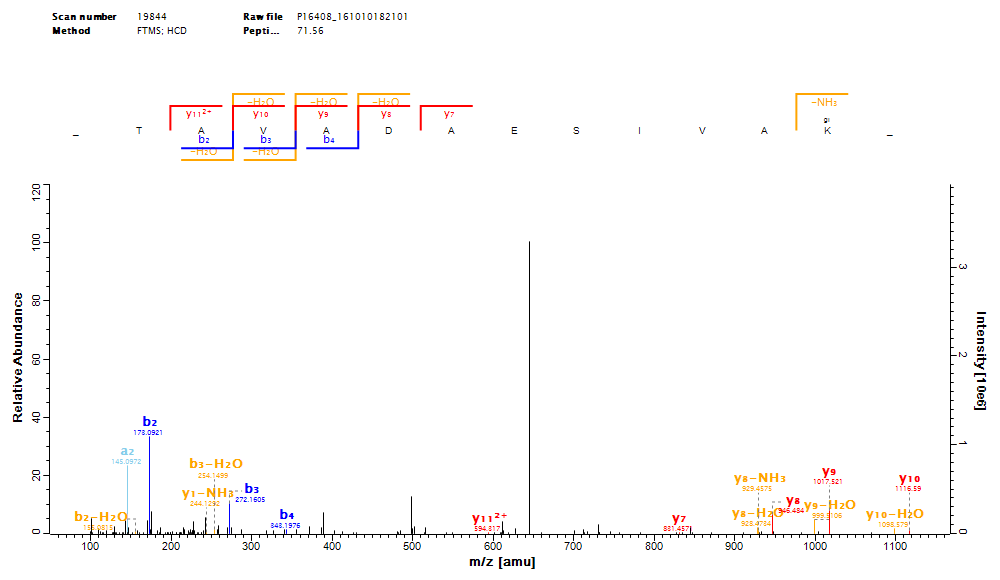


**A0A096UTL2**


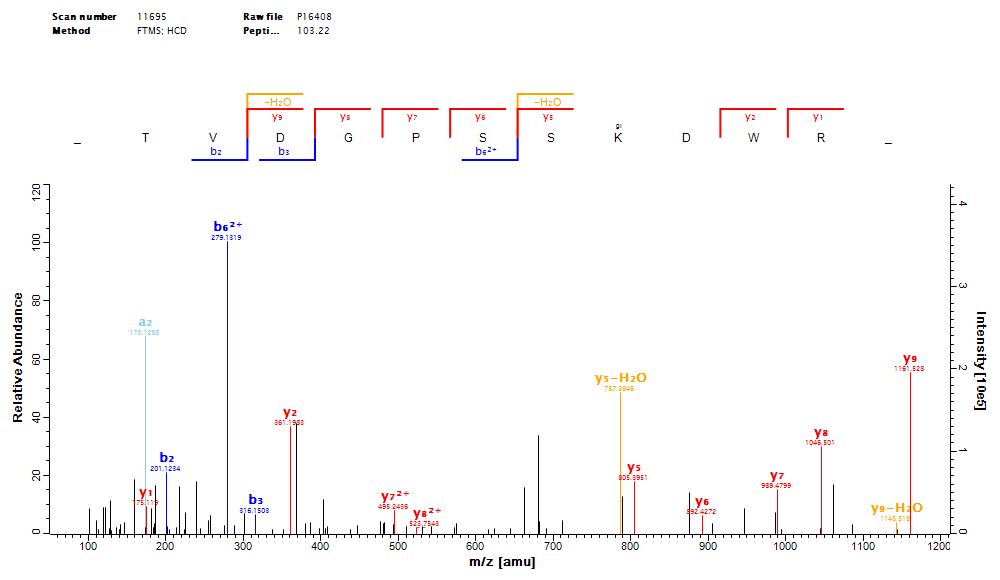


**
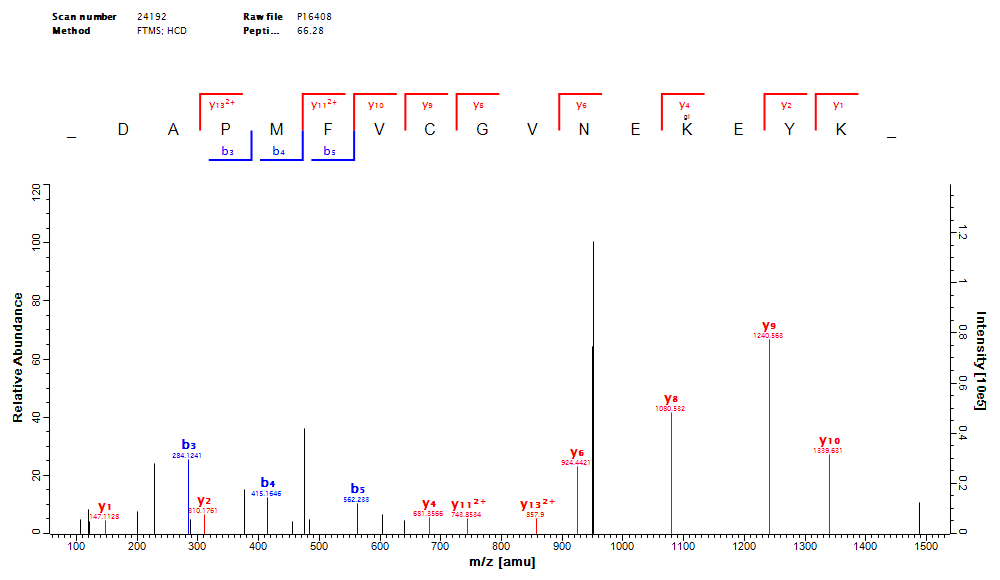
**

**
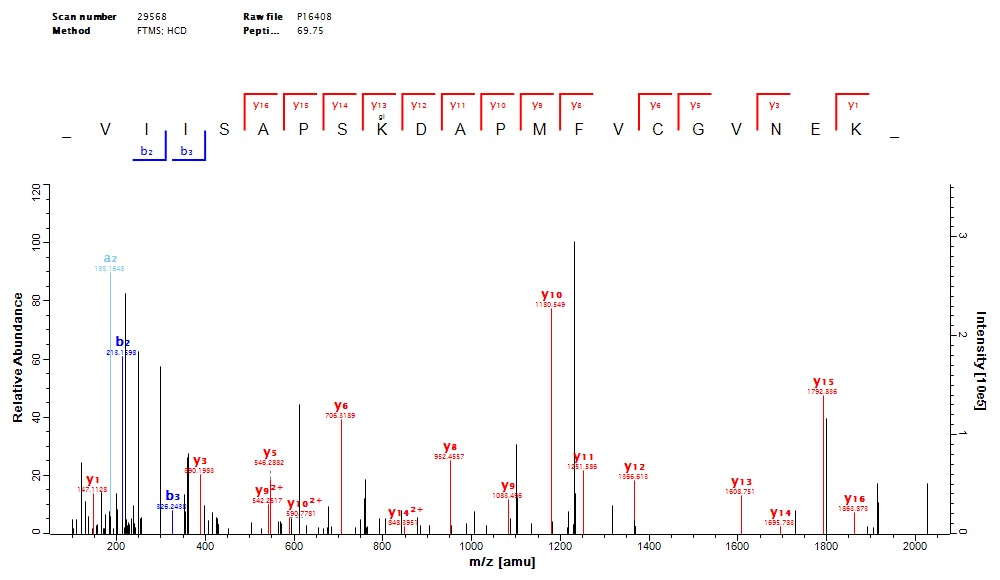
**

**P83970**

**
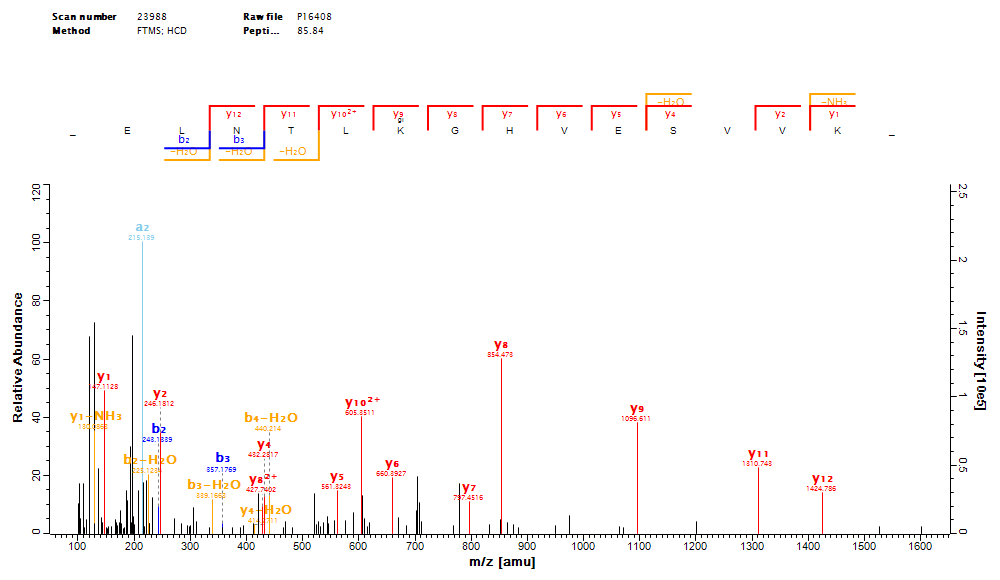
**

**
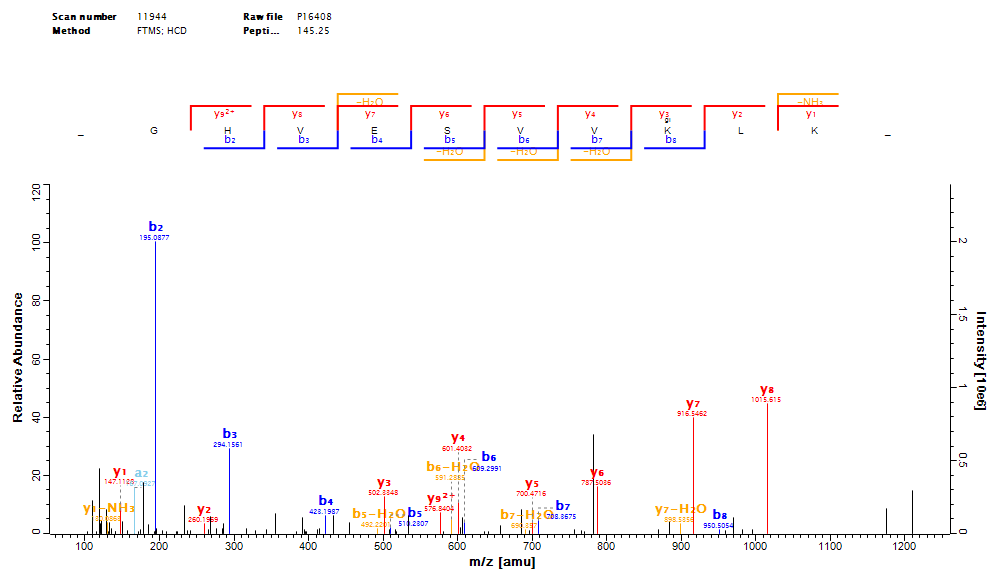
**

**
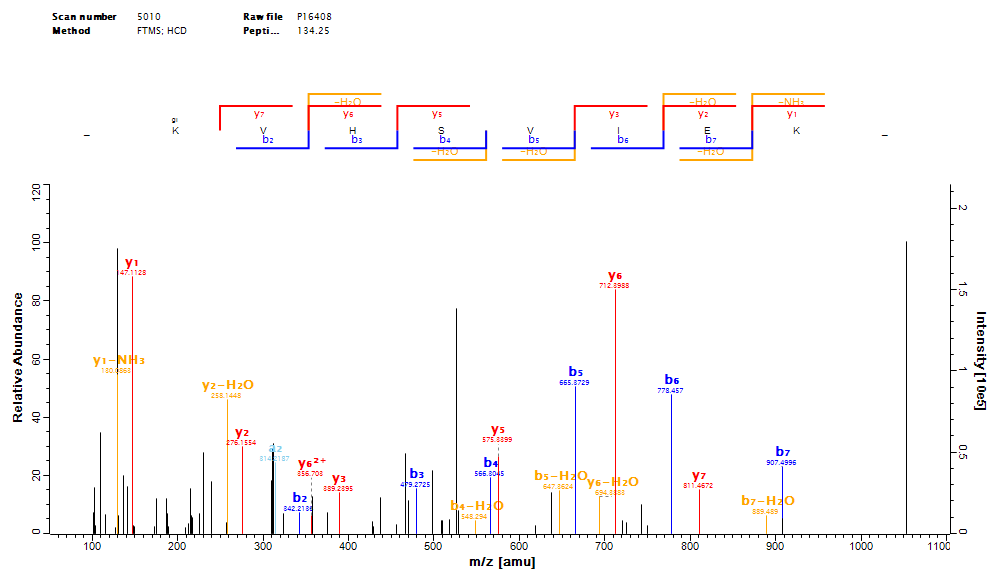
**

**
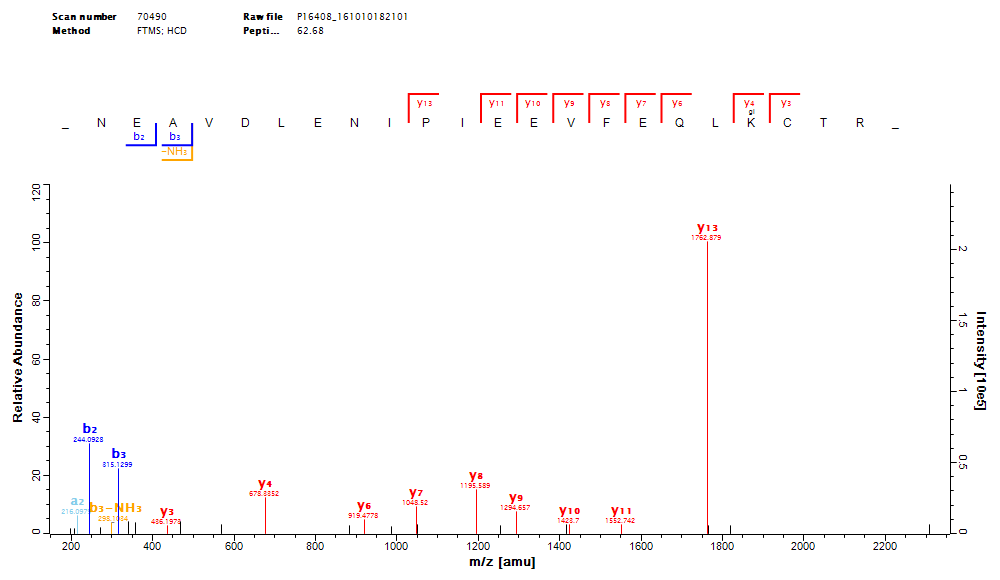
**

**
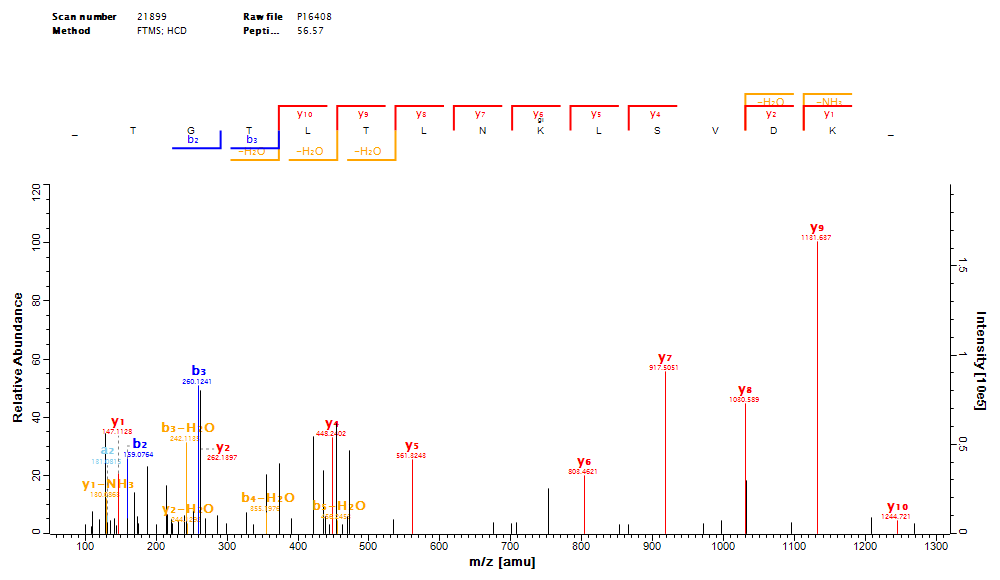
**

**W5A645**

**
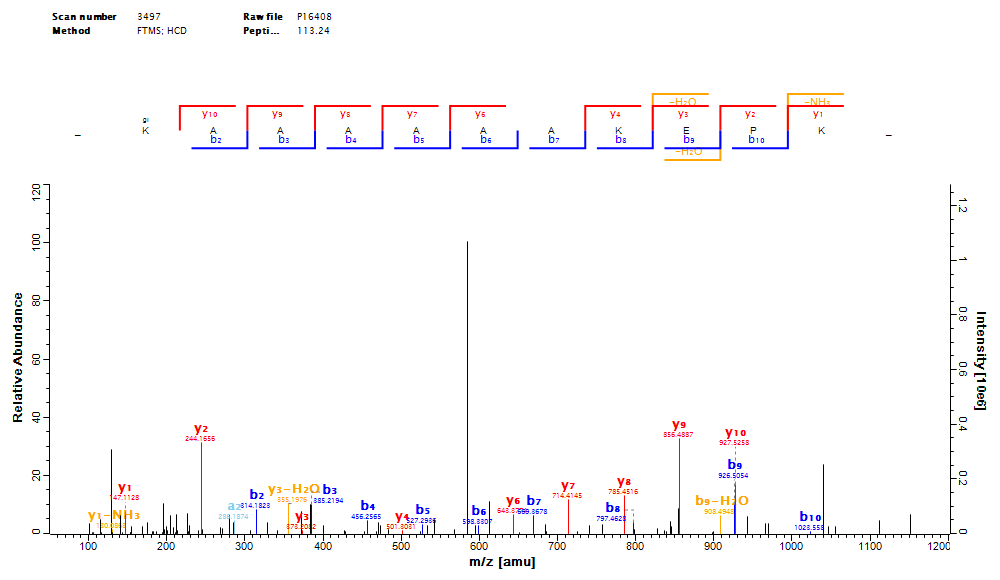
**
